# The prognostic value of neutrophil-to-lymphocyte ratio in adult carbapenem-resistant *Klebsiella pneumoniae* infection: a retrospective cohort study

**DOI:** 10.3389/fcimb.2024.1461325

**Published:** 2024-11-28

**Authors:** Zhongjie Wang, Renhua Li, Zhe Yuan, Zuli Zhang, Keli Qian

**Affiliations:** ^1^ Department of Infection Control, The First Affiliated Hospital of Chongqing Medical University, Chongqing, China; ^2^ Department of Respiratory and Critical Care Medicine, The First Affiliated Hospital of Chongqing Medical University, Chongqing, China

**Keywords:** carbapenem-resistant *Klebsiella pneumoniae*, risk factors, neutrophil-to-lymphocyte ratio, co-infection, prognosis

## Abstract

**Background:**

Systemic inflammatory indicators such as neutrophil-to-lymphocyte ratio (NLR) can effectively predict the prognosis of various inflammatory diseases. However, its prognostic effect on patients with carbapenem-resistant *Klebsiella pneumoniae* (CRKP) infection is little known. The objective of this study was to investigate the risk factors for mortality associated with CRKP infection and the clinical value of NLR in predicting prognosis in these patients.

**Methods:**

A total of 190 inpatients with CRKP infection from 1 January 2023 to 31 December 2023 were enrolled in this study, namely, 73 fatal cases and 117 survival cases in hospital. The medical data and examination results of these patients were collected. A logistic regression analysis was performed to assess the association between the NLR on the day of CRKP infection onset and all-cause mortality in hospital.

**Results:**

The overall mortality rate of patients with CRKP infection was 38.42% (73/190). Of the 190 patients, 91 were co-infected with carbapenem-resistant *Acinetobacter baumannii*/carbapenem-resistant *Pseudomonas aeruginosa* (CRAB/CRPA). Multifactor regression analysis confirmed that carbapenem exposure in the past 14 days, central line insertion, and chronic Foley catheter requirement were independent risk factors for carbapenem-resistant bacteria co-infection. The multivariate analysis shows that admission to an ICU, co-infection with CRAB/CRPA, and higher NLR were independent risk factors for the mortality in hospital, while appropriate treatment within 3 days was an independent protective factor. The area under the curve (AUC) of the NLR was 0.696, and the cutoff value of the NLR was 10.73.

**Conclusions:**

The NLR on the day of CRKP infection onset, admission to an ICU, and co-infection with CRAB/CRPA were identified as independent risk factors for all-cause mortality of patients with CRKP infection, while appropriate treatment within 3 days was recognized as an independent protective factor. The NLR serves as a conveniently accessible and independent prognostic biomarker for patients with CRKP infection.

## Introduction

Carbapenem-resistant Gram-negative bacilli such as carbapenem-resistant *Klebsiella pneumoniae* (CRKP), carbapenem-resistant *Pseudomonas aeruginosa* (CRPA), and carbapenem-resistant *Acinetobacter baumanii* (CRAB) are major pathogens of hospital-acquired infections, and are all contained in the bacterial pathogen priority list issued by the WHO in 2024 (World Health Organization (WHO), 2024). As one of the bacteria of the critical group, CRKP has widely spread around the world in the past 20 years and has also shown a rapid rising trend in Chinese medical institutions. According to the China Antimicrobial Surveillance Network, carbapenem resistance of *K. pneumoniae* has increased significantly from 3% to 26% between 2005 and 2023 (http://www.chinets.com/Data/GermYear).

The mortality rate of patients with CRKP infection was higher than that of patients with carbapenem-sensitive *Klebsiella pneumoniae* (CSKP) infection (Falagas et al., 2014; Budhram et al., 2020). Several studies have suggested that patients infected with CRKP have a mortality rate of up to 70% (Iredell et al., 2016; Han et al., 2022; Giacobbe et al., 2023). At present, the antimicrobial options for CRKP infection is still very limioted (Karampatakis et al., 2023). Meanwhile, in daily monitoring, we found that there were often cases in which multiple carbapenem-resistant bacteria were detected in one patient during the same hospitalization; in particular, CRKP and CRAB or CRPA were detected simultaneously. This phenomenon has also been reported in other studies. A study in the United States suggested that approximately 9% of patients had two or more different carbapenem-resistant negative bacteria co-colonized (Adediran et al., 2020). Another study found that over 30% of CRKP-positive patients were co-colonized with CRAB or CRPA (Sophonsri et al., 2023).

The treatment of carbapenem-resistant bacterial co-infections may be more complex, while the prognosis may be worse. Therefore, it is necessary to explore the risk factors for mortality associated with CRKP infection, especially co-infection of multiple carbapenem-resistant bacteria, to help clinicians to choose more appropriate empirical treatment and consequently improve the prognosis. However, studies on risk factors for mortality of patients with CRKP infection have been inconsistent; in addition, fewer studies have been conducted on co-infection with multiple carbapenem-resistant bacteria (Chen et al., 2022; Lv et al., 2022; Wu et al., 2022).

Leukocytes, neutrophils, or procalcitonins (PCTs) are usually used to reflect the level of inflammation in patients. However, these indicators can be influenced by many aspects; the accuracy of using one indicator alone to assess infection status is limited (Li et al., 2021). A growing number of research is combining these parameters to more effectively reflect inflammation levels. Neutrophil-to-lymphocyte ratio (NLR) was one of the important indicators used as an additional infection marker in clinical intensive care unit practice. Li et al. found that NLR was a risk factor for postoperative complication sepsis in patients with ureteral stones (Li et al., 2024). Zhang et al. observed that NLR first increased and then decreased during intracerebral hemorrhage, which had a high predictive value for pneumonia 30 days after surgery (Zhang et al., 2024).

Therefore, the primary objective of this study was to implore the risk factors of mortality associated with CRKP infection, especially the co-infection and mortality of multiple carbapenem-resistant bacteria, and secondly to evaluate NLR as a predictor of the prognosis of CRKP infection.

## Materials and methods

### Study design and data collection

**Figure 1:**
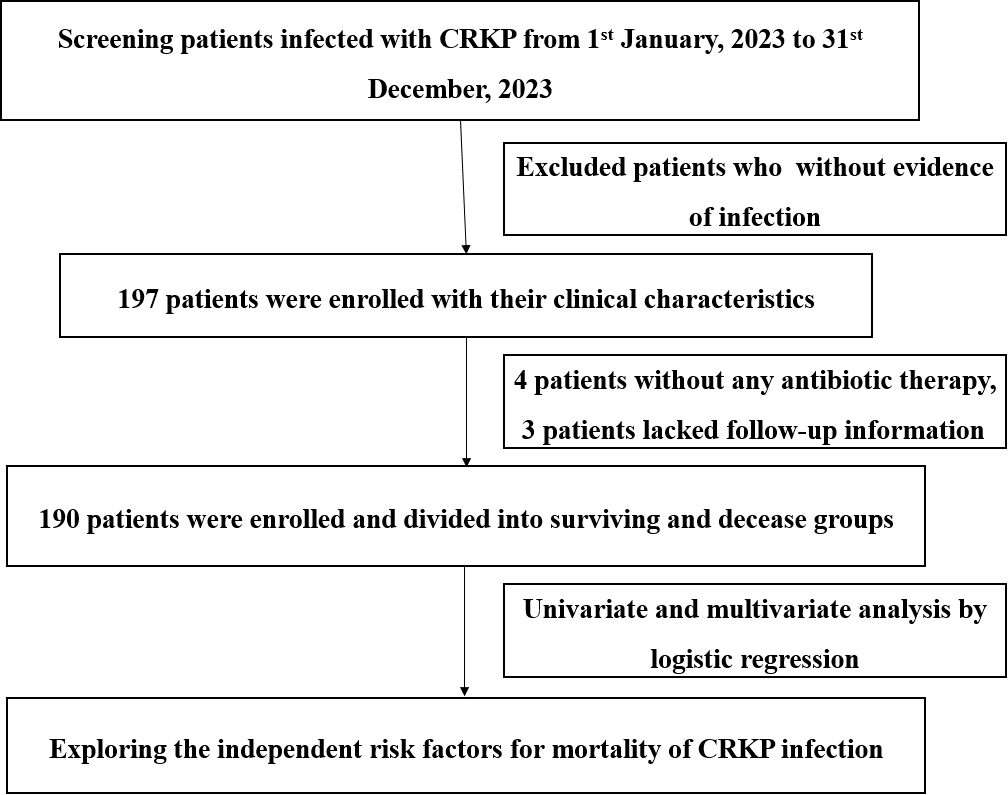
Flowchart of the study design.

This was a retrospective descriptive analysis conducted from 1 January 2023 to 31 December 2023 at a large tertiary hospital with 3,200 beds in Chongqing, China. The study was approved by the ethical research committee of The First Affiliated Hospital of Chongqing Medical University and was performed in accordance with the Declaration of Helsinki and its amendments. All data in this study were from medical records and stripped of identifying information. The predefined route of this study is shown in **Figure 1**.

We included patients with CRKP mono-infection and CRKP co-infection with CRAB or CRPA from the database with their medical records. Only the initial positive culture of target strains were included. The exclusion criteria were as follows: (i) patients aged 0–14 years, (ii) positive culture only without any evidence of infection; and (iii) medical records were unavailable for analysis.

All data for the same patient were collected by the same person, and another person checked the data entry to ensure accuracy. Gather information from medical records: age, gender, length of hospitalization, course of treatment [intensive care unit (ICU) stay, invasive procedures, and equipment], site of infection, antibiotics exposure, baseline diseases, and outcome. All blood laboratory results were obtained on the day of bacterial culture specimen collected.

### Study definitions

The onset of CRKP infection was defined as the collection date of a positive culture. Co-infection is defined based on the detection of ≥2 carbapenem-resistant bacteria in the same sample during hospitalization. A diagnosis of infection rather than colonization is confirmed by the clinician based on clinical signs and symptoms, imaging reports, and assessable clinical indicators. Carbapenem exposure meant that carbapenem antibiotics were administered for more than 48 h within the past 14 days. Appropriate treatments within 3 days were defined as administration of one or more antimicrobial agents proven effective by *in vitro* susceptibility testing within 72 h of definitive diagnosis of CRKP infection. All-cause mortality was defined as death from any cause during hospitalization.

### Statistical analysis

Statistical analysis was performed using IBM SPSS version 18.0 (IBM Corp., Armonk, NY, USA). Continuous variables are presented as mean ± standard deviation or median (interquartile range), Student’s *t*-test was used to compare normally distributed variables, and Mann–Whitney *U*-test was used to compare non-normally distributed variables. Categorical variables are presented as percentages, evaluated by chi-square or Fisher’s exact tests. Risk factors for mortality and co-infection were analyzed using logistic regression. Variables with *p* < 0.05 in the univariate analysis were included in a multivariate backward logistic regression analysis. *p* < 0.05 was considered to be statistically significant in multivariate logistic regression. The prediction accuracy was evaluated using the area under the receiver operating characteristic (ROC) curves.

## Results

### Characteristics of patients

From 1 January 2023 to 31 December 2023, a total of 197 patients met study inclusion criteria, but 4 patients did not undergo antibiotic therapy and 3 patients lacked follow-up information; thus, 190 patients were ultimately enrolled, of whom 73 died in hospital and 117 survived. The overall mortality rate was 38.42% (73/190). The clinical and demographic characteristics of cohort patients with CRKP infection are shown in **Table 1** according to the in-hospital survival status.

**Table 1:** Clinical and demographic characteristics of 190 patients with CRKP infection and risk factors for mortality in hospital.

	Total *N* = 190	Death in hospital *N* = 73	Surviving in hospital *N* = 117	*p*
Demographic				
Age	62.75 ± 17.22	67.03 ± 16.80	60.09± 17.01	0.007
Male, *n* (%)	136 (71.58)	51 (69.86)	85 (72.65)	0.679
Admission to an ICU, *n* (%)	84 (44.21)	63 (86.30)	21 (17.95)	<0.001
Duration of hospitalization in the ICU ≤ 3 days, *n* (%)	53 (27.89%)	5 (6.85%)	48 (41.03%)	<0.001
Total hospital stay, days (median, IQR)	50.00 (28.00, 80.00)	34.00 (20.00, 58.00)	57.00 (33.00, 87.00)	<0.001
Surgery, *n* (%)	76 (40.00)	23 (31.51)	53 (45.30)	0.059
Baseline disease				
Diabetes	30 (15.79)	13 (17.81)	17 (14.53)	0.547
Hypertension	39 (20.51)	14(19.18)	25(21.37)	0.854
Heart disease	15 (7.89)	12 (16.44)	3 (2.56)	0.001
Neurologic disease	55 (28.95)	15(20.55)	40(34.19)	0.049
Chronic nephritis	9 (4.74)	5(6.85)	4(3.42)	0.309
Immunosuppression	10 (5.26)	5(6.85)	5(4.27)	0.511
Pulmonary disease	29 (15.26)	16(21.92)	13(11.11)	0.061
Malignancy	33 (17.37)	12(16.44)	21(17.95)	0.846
Antimicrobial therapy				
Carbapenems exposure in past 2 weeks	97 (51.05)	39 (53.42)	58 (49.57)	0.605
Appropriate treatments within 3 days	131 (68.95)	32 (43.84)	99 (84.62)	<0.001
Infection situation				
Co-infection, *n* (%)	100 (52.63)	48 (65.75)	52 (44.44)	0.004
Pneumonia	126 (66.32)	52 (71.23)	74 (63.25)	0.274
Sepsis	29 (15.26)	14 (19.17)	15 (12.82)	0.300
Urinary tract infection	12 (6.32)	1 (1.37)	12 (6.32)	0.018
Abdominal infection	18 (9.47)	18 (9.47)	18 (9.47)	0.355
Intracranial infection	2(1.05)	0(0)	2(1.71)	0.524
Skin and soft tissue infection	3(1.58)	0(0)	3(2.56)	0.286
Use of medical device				
Mechanical ventilation, *n* (%)	113 (59.47)	59 (80.82)	54 (46.15)	<0.001
Central line insertion, *n* (%)	146 (76.84)	68 (93.15)	78 (66.67)	<0.001
Chronic Foley catheter requirement, *n* (%)	174 (91.58)	69 (94.52)	105 (89.74)	0.249
Laboratory variables from blood				
WBC (10^9^/L)	12.15 ± 6.48	15.26 ± 6.93	10.23 ± 5.38	<0.001
NEUT (10^9^/L)	10.01 ± 6.15	13.11 ± 6.75	8.10 ± 4.87	<0.001
LY (10^9^/L)	1.15 ± 1.29	1.18 ± 1.94	1.13 ± 0.65	0.82
PLT (10^9^/L)	232.99± 135.59	224.89± 154.68	238.02± 122.71	0.52
PT (s)	14.25 ± 3.65	15.11 ± 5.30	13.72 ± 1.90	0.036
A (g/L)	32.21 ± 4.79	31.29 ± 4.40	32.78 ± 4.95	0.038
TP (g/L)	61.95 ± 8.41	62.14 ± 8.20	61.84 ± 8.57	0.812
Cr (μmol/L)	80.59 ± 122.78	99.77 ± 129.95	68.79 ± 117.16	0.092
CRP (mg/L)	82.33 ± 83.77	107.28 ± 99.88	65.33 ± 66.10	0.005
PCT (ng/mL)	12.99 ± 36.18	16.43 ± 45.32	10.75 ± 28.74	0.312
Blood glucose (mmol/L)	7.38 ± 3.63	9.64 ± 5.26	6.36 ± 1.91	0.008
TC (mmol/L)	3.71 ± 1.19	3.41 ± 1.28	3.88 ± 1.12	0.045
TG (mmol/L)	1.63 ± 1.06	1.73 ± 1.30	1.57 ± 0.91	0.463
NLR	15.88 ± 22.23	26.74 ± 32.27	9.25 ± 7.01	<0.001

ICU, intensive care unit; WBC, white blood cell; NEUT, neutrophil; LY, lymphocyte count; PLT, platelet; PT, prothrombin time; A, albumin; TP, total protein; Cr, creatinine; CRP, C-reactive protein; PCT, procalcitonin; TC, total cholesterol; TG, triglyceride; NLR, neutrophil-to-lymphocyte count ratio; CRKP, carbapenem-resistant *Klebsiella pneumoniae.*

### Characteristics of patients with co-infection and mono-infection

Of 146 patients, 99 had CRKP mono-infection and 91 had co-infection with CRKP and CRPA or CRAB. Of those with co-2infections, 62.64% (57/91) had CRKP and CRAB, 12.09% (11/91) had CRKP and CRPA, while 25.27% (23/91) had all three pathogens (CRKP, CRPA, and CRAB). There was no difference in the proportion of patients admitted to ICU and who underwent surgery between the two groups. However, patients in the co-infected group had higher rates of use of medical devices, including mechanical ventilation (*p* = 0.003), central line insertion (*p* = 0.002), and chronic Foley catheter requirement (*p* < 0.001). The sites of infection differed between the two groups (**Table 2**). The predominant source of infection in both groups was the respiratory tract, but the proportion was higher in the co-infection group (*p* = 0.004), while the urinary tract infection was more common in patients with mono-infection (*p* = 0.035). Antimicrobial exposure before the onset of CRKP infection was compared between the two groups. Notably, a higher proportion of co-infected patients were exposed to carbapenems, and a lower proportion were exposed to beta-lactam/beta-lactamase inhibitor than the mono-infection group. Meanwhile, meropenem was found to be the dominant carbapenem antibiotic, and more than 80% of patients in both groups were on combination therapy.

**Table 2:** Comparison of co-infection and mono-infection. ICU, intensive care unit. * Carbapenem was used in monotherapy.

	Co-infection, *N* = 91	Mono-infection, *N* = 99	*p*-value
Therapeutic processes before infection			
Admission to an ICU, *n* (%)	44 (48.35)	40 (40.40)	0.307
Duration of hospitalization in the ICU ≤ 3 days, *n* (%)	15 (16.48%)	38 (38.39%)	0.001
Surgery, *n* (%)	36 (39.56)	40 (40.40)	0.534
Mechanical ventilation, *n* (%)	64 (70.33)	49 (49.50)	0.003
Central line insertion, *n* (%)	79 (79.80)	67 (67.68)	0.002
Chronic Foley catheter requirement, *n* (%)	90 (98.90)	84 (84.85)	<0.001
Infection situation			
Pneumonia, *n* (%)	70 (76.92)	56 (56.57)	0.004
Sepsis, *n* (%)	9 (9.89)	20 (20.20)	0.068
Urinary tract infection, *n* (%)	2 (2.20)	10 (10.10)	0.035
Abdominal infection, *n* (%)	7 (7.69)	11 (11.11)	0.466
Intracranial infection, *n* (%)	0 (0)	2 (2.02)	0.498
Skin and soft tissue infection, *n* (%)	3 (3.30)	0 (0)	0.108
Antimicrobial exposure in the past 14 days			
Carbapenems, *n* (%)	71 (78.02)	42 (42.42)	<0.001
Meropenem	58	32	0.483
Imipenem	13	10	
Monotherapy*	9	4	0.612
Beta-lactam/beta-lactamase inhibitor, *n* (%)	57 (62.64)	88 (88.89)	<0.001
Tigecycline, *n* (%)	5 (5.49)	6 (6.06)	0.558
Fluoroquinolones, *n* (%)	10 (10.99)	10 (10.10)	0.514
Sulfamethoxazole, *n* (%)	6 (6.59)	7 (7.07)	0.564
Aminoglycosides, *n* (%)	8 (8.79)	9 (9.09)	0.583

### Risk factors associated with CRKP co-infection with CRAB/CRPA

By multivariable logistic regression analysis, the following clinical variables were identified for our cohort as independent risk factors that were significantly associated with co-infection: carbapenem exposure in past 14 days, central line insertion, and chronic Foley catheter requirement (**Table 3**).

**Table 3:** Multivariate analysis of risk factors for CRKP co-infection with CRAB/CRPA.

	Exp (B)	95% CI Exp (B)	*p*-value
Central line insertion	2.688	1.24–5.825	0.012
Chronic Foley catheter requirement	15.793	1.975–126.314	0.009
Carbapenems exposure in the past 14 days	0.472	0.243–0.917	0.027

CRKP, carbapenem-resistant *Klebsiella pneumoniae*; CRAB, carbapenem-resistant *Acinetobacter baumanii*; CRPA. carbapenem-resistant *Pseudomonas aeruginosa*.

### Risk factors associated with mortality of CRKP infection

**Figure 2:**
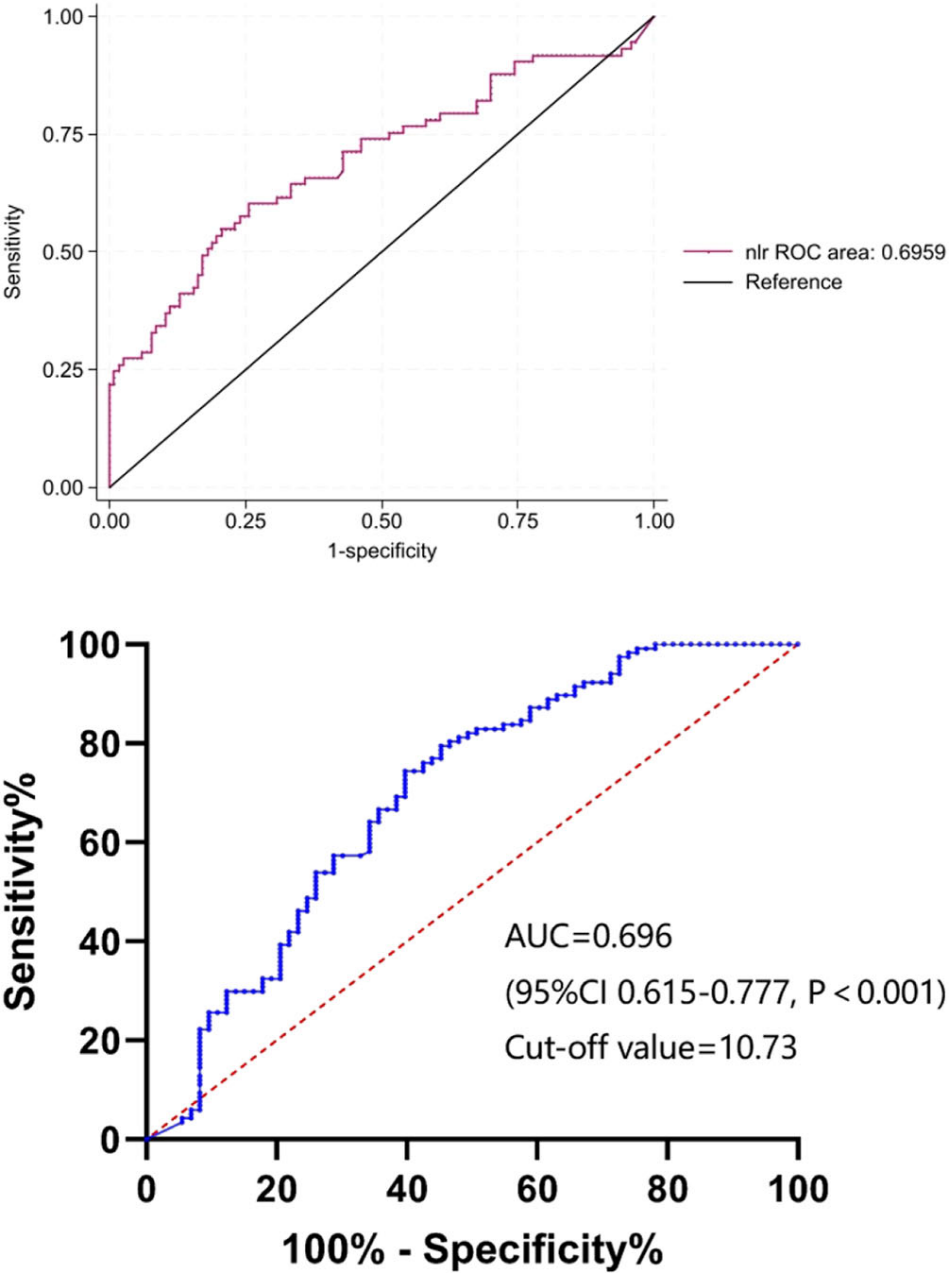
The ROC curves of NLR. ROC, receiver operating characteristic; NLR, neutrophil-to-lymphocyte count ratio; AUC, area under the curve.

To identify the potential risk factors for mortality of CRKP infection, we conducted univariate analyses between the surviving and deceased groups. The results (**Table 2**) revealed that admission to an ICU (*p* < 0.001), co-infection with CRAB/CRPA (*p* = 0.005), use of mechanical ventilation (*p* < 0.001), central line insertion (*p* < 0.001), higher white blood cell (*p* = 0.009), higher neutrophil (*p* = 0.002), higher prothrombin time (*p* = 0.004), lower albumin (*p* = 0.005), higher C-reactive protein (*p* = 0.004), higher blood glucose (*p* = 0.007), and higher NLR (*p* < 0.001) were more likely to be associated with mortality in hospital. In addition, the multivariate analysis (**Table 4**) shows that admission to an ICU (*p* < 0.001), co-infection with CRAB/CRPA (*p* = 0.005), and higher NLR (*p* < 0.001) were independent risk factors for mortality in hospital, while appropriate treatment within 3 days (*p* < 0.001) was an independent protective factor. The ROC curves of the NLR and in-hospital mortality are shown in **Figure 2**, and the area under the curve (AUC) was 0.696 (95% CI 0.615–0.777, *p* < 0.001); the NLR value with the highest Youden index was also the cutoff value of the NLR, which was 10.73.

**Table 4:** Multivariate logistic regression analysis of risk factors for mortality in hospital of patients with CRKP infection.

	Univariate analysis			Multivariate analysis		
	Exp (B)	95% CI Exp (B)	*p*-value	Exp (B)	95% CI Exp (B)	*p*-value
Admission to an ICU	0.035	0.015–0.079	<0.001	0.034	0.012–0.091	<0.001
Duration of hospitalization in the ICU ≤ 3 days, *n* (%)	0.106	0.040–0.282	<0.001			
Co-infection with CRAB/CRPA	0.417	0.227–0.763	0.005	0.141	0.024–0.841	0.032
Appropriate treatments within 3 days	7.047	3.561–13.946	<0.001	7.928	2.627–20.271	<0.001
Mechanical ventilation	0.203	0.102–0.404	<0.001			
Central line insertion	0.147	0.055–0.394	<0.001			
WBC	0.939	0.895–0.984	0.009			
NEUT	0.923	0.877–0.972	0.002			
PT	0.806	0.696–0.932	0.004			
A	1.101	1.03–1.176	0.005			
CRP	0.994	0.989–0.998	0.004			
Blood glucose	0.801	0.682–0.942	0.007			
NLR	0.933	0.902–0.964	<0.001	0.968	0.935–1.001	0.036

CRKP, carbapenem-resistant *Klebsiella pneumoniae*; ICU, intensive care unit; CRAB, carbapenem-resistant *Acinetobacter baumanii*; CRPA. carbapenem-resistant *Pseudomonas aeruginosa*; WBC, white blood cell; NEUT, neutrophil; PT, prothrombin time; A, albumin; CRP, C-reactive protein; NLR, neutrophil-to-lymphocyte count ratio.

### Co-infection with CRAB/CRPA; a higher NLR may increase 28-day mortality

According to survival at 28 days after the onset of CRKP infection, we divided the patients who died into those who died within 28 days and those who died after 28 days. After univariate and multifactorial regression analysis, it was found that co-infection with CRAB/CRPA and high NLR at the onset of CRKP infection were risk factors for death within 28 days (**Table 5**).

**Table 5:** Multivariate logistic regression analysis of risk factors for 28-day mortality of patients with CRKP infection.

	Exp (B)	95% CI Exp (B)	*p*-value
Co-infection with CRAB/CRPA	6.415	1.526–26.978	0.011
NLR	1.204	1.071–1.353	0.002

CRKP, carbapenem-resistant *Klebsiella pneumoniae*; CRAB, carbapenem-resistant *Acinetobacter baumanii*; CRPA, carbapenem-resistant *Pseudomonas aeruginosa*; NLR, neutrophil-to-lymphocyte count ratio.

## Discussion

CRKP infection is an urgent public health problem. Because of the limited treatment availability coupled with the rapid spread of CRKP infection, the treatment of patients after infection is difficult, the case fatality rate is high, and the length of hospital stay and the cost are significantly increased, which increases the burden of patients and consumes more medical resources (Kohler et al., 2017). Owing to the abuse of antibiotics, environmental transmission, quorum sensing, and host adaptive response, CRKP co-infection with other carbapenem-resistant bacteria further increased the difficulty of clinical treatment (Marchaim et al., 2011; Sophonsri et al., 2023).

In the surveillance data for 2023, we found that the incidence of CRKP and CRAB/CRPA co-infection was significantly higher. A recent study has suggested that *P. aeruginosa* and *K. pneumoniae* are common co-isolates of *Acinetobacter baumannii*, which is consistent with our findings (Karakonstantis et al., 2022). In this study, 48.35% of co-infected patients were admitted to the ICU. A previous study has shown that 31.3% of the high-frequency contact surface cultures in the ICU bed units had one or more CRKP positive (Yan et al., 2019). At the same time, there was also a correlation between co-infection and ICU stay time. We found that the rate of co-infection was relatively lower in an ICU stay of less than 3 days (co-infection 16.48% vs. mono-infection 38.39%, *p ≤* 0.001).

An investigation into the outbreak of multidrug-resistant bacterial hospital infections in the ICU found that environmental contamination played an important role in some instances. Drug-resistant bacteria that are airborne or transmitted through contact may lead to healthcare-acquired infections or colonization, and even co-infections with multidrug-resistant carbapenemase-producing bacteria (Marchaim et al., 2012). Compared with CRKP mono-infection patients, co-infected patients were more likely to undergo invasive procedures, and we found that up to 98.90% of the co-infected patients in this study required urinary catheters and that 70.33% and 79.8% required the use of ventilators and central line insertion, respectively. Consistent with the results of another study, it was suggested that patients with co-infection were severely debilitated and lacked a functional state of activities of daily living, especially incontinence (Sophonsri et al., 2023). Simultaneously, these invasive procedures also increased the risk of patients contracting or colonizing multidrug-resistant bacteria. The respiratory tract was the main infection site, and the proportion of patients in the co-infected group were higher, which may be related to the higher utilization rate of ventilators in the co-infected group.

A higher proportion of patients with co-infections had been exposed to carbapenem antibiotics within 14 days prior to the onset of CRKP infection. Carbapenem overexposure may select carbapenem-resistant organisms through different mechanisms, such as porin deletion or mutation, efflux pump overexpression, and upregulation of carbapenem resistance genes such as blaKPC (Tsao et al., 2018; Zou et al., 2018). Meanwhile, all patients were exposed to antibiotics within 14 days, including beta-lactam/beta-lactamase inhibitor and fluoroquinolones in addition to carbapenems. The complex underlying medical conditions of our patients likely required the use of broad-spectrum agents to provide adequate coverage but also predisposing them to infections caused by multidrug-resistant organisms. By multivariate analysis, clinical features identified to be significantly associated with co-infection in our study cohort were use of central line insertion, chronic Foley catheter requirement, and carbapenem exposure in the past 14 days.

This study observed an all-cause mortality of 38.42% in patients with CRKP infection, similar to the mortality reported in previous studies (Chen et al., 2021; Barbosa et al., 2022). The mean age of patients in the hospital death group was higher than that in the hospital survival group, while the total hospital stay was shorter. In the hospital death group, admission to an ICU, neurologic disease, the use of medical device (mechanical ventilation, central line insertion, and chronic Foley catheter requirement), and co-infection with CRAB/CRPA were significantly more common than in the hospital survival group. It was found that patients in the death group whose duration of hospitalization in the ICU was ≤3 days were significantly less than those in the surviving group, suggesting that the patients in the death group may have more complex and severe diseases and were more likely to have co-infection during their stay in the ICU, which aggravated their disease. We also found that WBC, neutrophils, CRP, and NLR were higher; albumin was lower; and blood glucose and total cholesterol levels were higher in the hospital death group.

In the multivariate logistic regression analysis, admission to an ICU and co-infection with CRAB/CRPA were independent risk factors for all-cause mortality of CRKP infection patients. Previous studies suggest that age is a restrictive factor for ICU admission and determines treatment intensity (Tian et al., 2016). Among patients admitted to the ICU, there is an increased risk of antibiotic-resistant microbial infections and increased mortality due to comorbidities, critical conditions, frequent use of antimicrobials, and invasive procedures. Because of the age-related decline in immune function, age-related changes in organ structure and function, malnutrition, and comorbidities, elderly patients are susceptible to infections (Çölkesen et al., 2023), increasing the likelihood of admission to the ICU (Zhou et al., 2023). Age was not found to be an independent risk factor for morbidity, and this may be due to the fact that the study enrolled patients who were primarily elderly, with an average age of over 60 years.

Gender was not associated with mortality, but it is interesting to note that 71.58% of patients with CRKP infection were male. This is consistent with the findings of other literatures, which show that the proportion of male patients with CRKP infection is relatively high (Wang et al., 2022). One possible reason is that the common infection site of CRKP in China is the respiratory tract, and it is well known that men are more likely to develop respiratory tract infection due to smoking.

Because of the failure to implement early and rapid diagnosis, many patients were initially treated with inappropriate antibiotics, which may cause several serious comorbidities such as infectious shock, further increasing mortality. In this study, multivariate regression analysis results suggested that appropriate treatment within 3 days was a protective factor for mortality from CRKP infection. As mentioned earlier, carbapenem exposure increased the risk of co-infection. These data showed that inappropriate antibiotic exposure not only increased the risk of carbapenem-resistant bacteria infection but also increased mortality. Thus, a rational use of antibiotics can contribute to the decrease in morbidity and mortality associated with CRKP infection.

Studies have confirmed that early and appropriate anti-infective therapy can effectively reduce the mortality of CRKP infection (Kohler et al., 2017). Hence, exploring the prognostic value of potential biomarkers early on is important. A suitable biomarker must provide additional information to what is presently available; it should be able to predict outcomes or evaluate the efficacy of treatment, and it should be immediately available and cost-effective. In clinical practice, leukocytes, neutrophils, or PCTs are usually used to reflect the level of inflammation in patients. However, these indicators’ infection status is limited. As a reaction product in the acute phase, CRP increases when inflammation, infection, or tissue damage occurs in the body (Sproston and Ashworth, 2018; Tan et al., 2019), while PCT is also not a perfect marker of inflammation, as it can be elevated in any cellular injury, whether direct tissue injury or non-infectious ischemia–reperfusion injury, such as myocardial infarction or cancer, which can lead to misdiagnosis by clinicians (Paudel et al., 2020). Univariate analysis revealed that CRP was a prognostic factor in patients with CRKP infection in this study, but after multivariate analysis, we found that CRP did not effectively predict the prognosis of patients with CRKP infection.

Neutrophils is the first line of defense against bacterial infections, and they rapidly recruit and phagocytose to kill pathogenic microorganisms after bacterial infections. At the same time, lymphocyte apoptosis is accelerated, leading to immune system suppression and multiorgan dysfunction (Kobayashi et al., 2018). In some studies, NLR has been observed to be more effective than conventional inflammatory biomarkers in adult diseases such as community-acquired pneumonia (De Jager et al., 2012) and sepsis (Huang et al., 2020). This study indicated the NLR was an independent risk factor for all-cause mortality of CRKP infection patients. We conducted this study to evaluate NLR as a predictor of the prognosis of patients infected with CRKP, along with other biomarkers and therapy strategies. We found that NLR on the day of CRKP infection onset could be an independent risk factor for all-cause mortality and 28-day mortality. To the best of our knowledge, this is the first study to explore the value of the NLR in predicting all-cause mortality of patients with CRKP infection. It suggests that NLR can be used as a useful and economical marker with the function of indicating the severity of CRKP infection in patients and whether anti-infection treatment is appropriate for patients.

There are several limitations to this study. First, this was a single-center retrospective study, which may have introduced bias in data interpretation. Second, owing to the limitation of the retrospective study, we did not perform a dynamic analysis of NLR in patients with CRKP infection. Third, the ability to capture data pertinent to outside hospitalizations such as antimicrobial administration as well as readmissions to outside institutions also poses limitations to the completeness of data collection.

## Conclusion

Together, we observed that co-infection with CRKP and another carbapenem-resistant pathogen significantly increased morbidity and healthcare burden. Our study revealed that NLR on the day of CRKP infection onset, admission to an ICU, and co-infection with CRAB/CRPA were independent risk factors for all-cause mortality of patients with CRKP infection, while appropriate treatment within 3 days was an independent protective factor.

## Data Availability

The original contributions presented in the study are included in the article/supplementary material. Further inquiries can be directed to the corresponding author.
